# Deciphering Succession and Assembly Patterns of Microbial Communities in a Two-Stage Solid-State Fermentation System

**DOI:** 10.1128/Spectrum.00718-21

**Published:** 2021-09-22

**Authors:** Dengjin Shen, Hongye Shen, Qiang Yang, Shenxi Chen, Yaohao Dun, Yunxiang Liang, Jinshui Zheng, Shumiao Zhao

**Affiliations:** a State Key Laboratory of Agricultural Microbiology, College of Life Science and Technology, Huazhong Agricultural University, Wuhan, China; b Hubei Provincial Key Laboratory for Quality and Safety of Traditional Chinese Medicine Health Food, Jing Brand Company, Ltd., Daye, China; c Hubei Key Laboratory of Agricultural Bioinformatics, Huazhong Agricultural University, Wuhan, China; University of Minnesota

**Keywords:** *Xiaoqu* liquor, community succession, turnover, nestedness, community assembly

## Abstract

Although the importance of microbiota in the natural environment and in industrial production has been widely recognized, little is known about the formation and succession patterns of the microbial community, particularly secondary succession after disturbance. Here, we choose the *Xiaoqu* liquor brewing process as an experimental model in which sorghum grains were first aerobically saccharified and then anaerobically fermented after being stirred and acidified to explore multistage community succession patterns. We analyzed microbial composition, physicochemical factors, and metabolites of brewing grains inoculated with two different starters, pure starter and traditional starter, respectively. Two groups showed similar succession patterns where the saccharification microbiota was mainly derived from starters, while environmental microorganisms, mainly *Lactobacillaceae* and *Saccharomyces*, dominated the fermentation microbiota regardless of the original saccharification community composition. Species replacement shaped the bacterial community, while species replacement and loss both contributed to fungal community succession in both groups. Grain acidification and hypoxia led to the succession of bacterial and fungal communities during fermentation, respectively. Despite inoculation with starters containing different microorganisms, similar microbial communities during the fermentation stage of the two groups exhibited similar metabolite composition. However, higher abundance of *Rhizopus* in the saccharification of the pure starter group led to more alcohols, while higher abundance of *Monascus* and *Saccharomycopsis* in the traditional starter group promoted acid and ester metabolism. These results revealed the microbial succession patterns of two-stage liquor brewing and its influence on flavor metabolism, which could be used to regulate the microbial community in food fermentation to further promote the modernization of the fermented food industry.

**IMPORTANCE** Revealing formation and assembly mechanisms of microbiota can help us to understand and further regulate its roles in the ecosystems. The *Xiaoqu* liquor brewing system is a tractable microbial ecosystem with low complexity. This two-stage microbial ecosystem can be used as an experimental model to analyze the multistage temporal succession pattern of microbial communities. Our results demonstrated the dynamic composition and succession pattern of a microbial community in the two-stage liquor brewing system. The results also revealed the microbial origins determining community composition, the ecological processes dominating microbial community succession patterns, the determinants affecting microbial community successions, and the effect of microbial community changes on metabolite synthesis. Overall, our study not only provides an insight into multistage succession patterns of microbial communities in liquor brewing systems but also provides reference for optimizing the quality of fermented products, which will be helpful to understand the succession patterns of microbial communities in other natural ecosystems.

## INTRODUCTION

With the rapid development of next-generation sequencing technology, our knowledge of the composition and dynamics of complex microbial communities from multiple ecosystems has greatly advanced (1, 2). Biotic and/or abiotic factors have been extensively reported to affect community assembly and stability to different extents (3). Disturbances such as antibiotic treatment (4) and wildfire (5, 6) can significantly affect the structure of microbial communities. However, sophisticated mechanisms behind the formation and development of microbial community structures in natural ecosystems need to be further understood. Natural habitats such as soil and aquatic environments normally carry hundreds to thousands of microbial species, most of which are uncultured (7–9). These complex ecosystems, due to their nontraceability, are not ideal experimental models for studying the development of microbial communities (10). Fermented food, by contrast, is a relatively simple, reproducible, and diverse seminatural ecosystem that allows us to easily monitor community changes on both spatial and temporal scales. As a tractable ecosystem, fermented food can serve as an ideal experimental microbial model to explore the mechanisms of microbial community formation (11). High resource availability of fermented food ecosystems can induce the rapid development of microbial communities within a short period (12). Additionally, fermented foods are usually manufactured in open environments. Environmental microorganisms thus could play an important role in shaping the microbial composition in fermented food microbial ecosystems (13, 14). These characteristics make fermented food an ideal *in situ* model for analyzing the temporal succession of microbial composition (13, 15–17). Additionally, the flavor compounds metabolized by the microbial community determine the quality of fermented foods. Optimization of the functional microbial community in the production of fermented foods contributes to the production of high-quality products (18). Demand for the regulation of the fermentation functional microbiota has promoted attempts to explore the succession mechanism of the microbial community involved in fermented food production (19).

Baijiu is a typical Chinese fermented alcoholic beverage. More than 4 billion liters are consumed annually by Chinese (20). Unlike other distilled spirits, baijiu is produced through solid-state fermentation (SSF) or semi-SSF. Briefly, *Jiuqu* (similar to Koji) containing multiple functional microorganisms is used as a brewing starter and mixed with raw materials, such as sorghum, rice, corn, and wheat. The mixtures are further saccharified, fermented, and distilled under solid-state environments. Baijiu is normally divided into three typical categories: *Daqu* baijiu, *Xiaoqu* baijiu, and *Fuqu* baijiu (21). Different from the simultaneous saccharification and fermentation processes of *Daqu* baijiu in anaerobic environments, the brewing processes of *Xiaoqu* baijiu adopted separated hydrolysis (saccharification) and fermentation (SHF) (22). Raw materials are first placed in an open environment for aerobic saccharification, mechanically stirred and acidified by mixing with high-acidity distilled grains, and eventually transferred into the fermenters for anaerobic fermentation. The microorganisms involved in the brewing process are exposed to two completely different environments and an external disturbance, making the *Xiaoqu* baijiu ecosystem a desirable experimental model for simultaneously investigating both primary succession and secondary succession of a microbial community after disturbance.

Here, we choose the *Xiaoqu* liquor brewing system as an experimental model and designed an *in situ* experiment in a modern distillery to explore the multistage temporal microbial community succession patterns. Brewing experiments of *Xiaoqu* liquor inoculated with two different starters were performed. Assembly and dynamics of the microbial community during the brewing process were investigated by amplicon sequencing. The effects of different ecological processes on community assembly were evaluated by null model-based analysis. Additionally, a targeted metabolomics analysis was used to monitor the dynamic changes of flavor substances during the brewing processes. This study aims to characterize the dynamic microbial composition during the brewing process of *Xiaoqu* liquor, reveal the mechanisms and determinants of multistage community successions, and provide a paradigm for studying microbial community successions in other natural ecosystems. Moreover, understanding the microbial community composition and dynamic change patterns during the production of *Xiaoqu* liquor is also conducive to improving the quality and yield of alcoholic beverages.

## RESULTS

### Dynamics of the physicochemical parameters during the brewing process.

**FIG 1:**
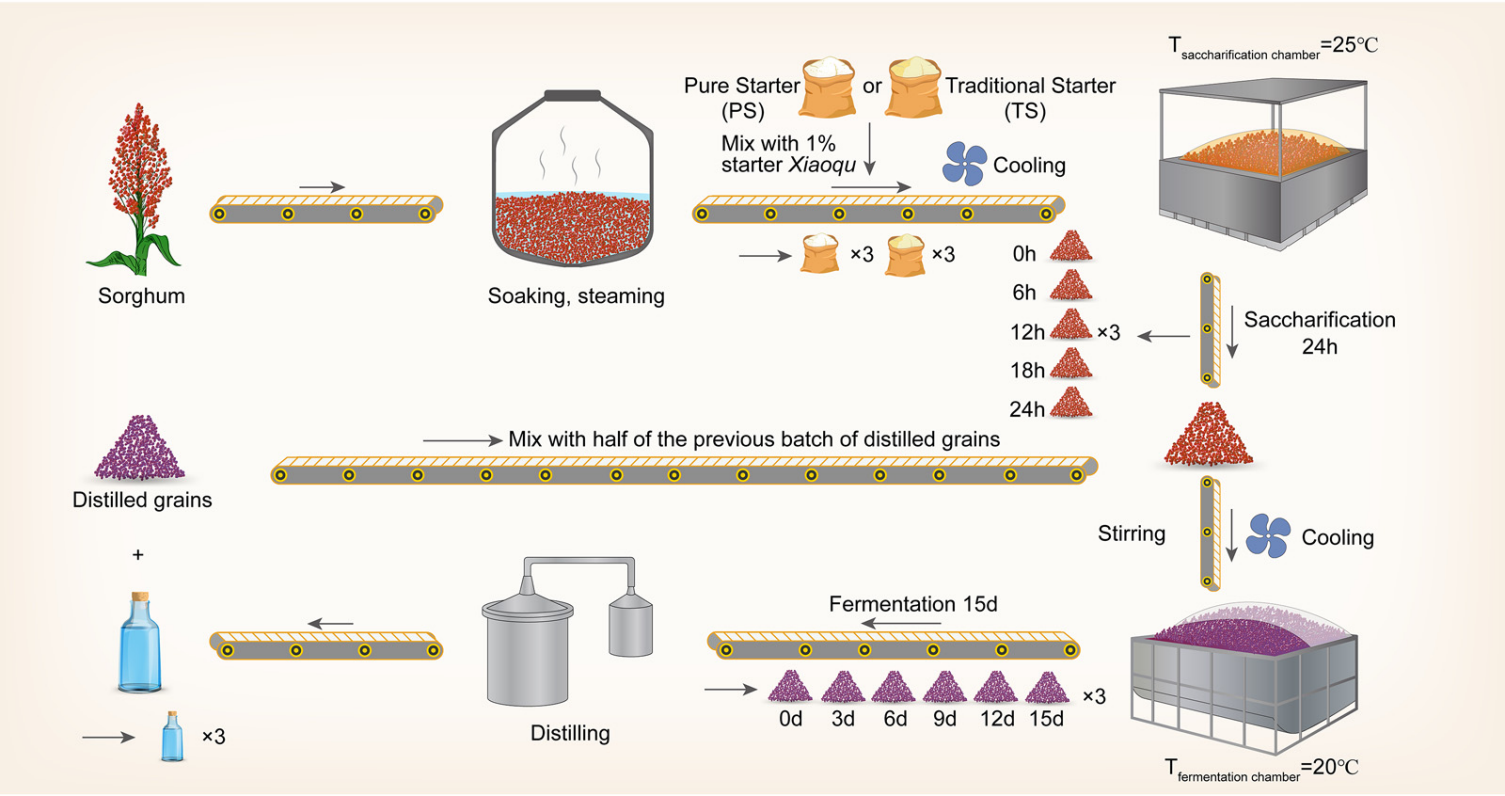
Schematic of the brewing process and sampling procedures of Chinese *Xiaoqu* light-flavor liquor. Starter powder, brewing grains, and fresh liquor were sampled in triplicate from pure starter (PS) batch and traditional starter (TS) batch. Grain samples were named by the combined information of the starter batch (P for PS batch, T for TS batch), brewing stage (SAC for saccharification stage, FER for fermentation stage), and sampling time points. Time units for saccharification and fermentation are indicated by “h” (hours) and “d” (days), respectively.

A total of 30 saccharified grains and 36 fermented grains from three separate batches in two groups inoculated with 2 different starters, pure starter (PS) and traditional starter (TS), were sampled for the analysis of physicochemical parameters during the brewing process (Fig. 1). Detailed information about the amplicon sequencing reads and processing of bacterial 16S V4 and fungal internal transcribed spacer 2 (ITS2) are listed in Table S1 and S2 in the supplemental material, respectively. Alpha rarefaction curves showed that the sequencing depth was sufficient; thus, the amplicon sequencing results could represent the real microbial community compositions (Fig. S1).

**FIG 2:**
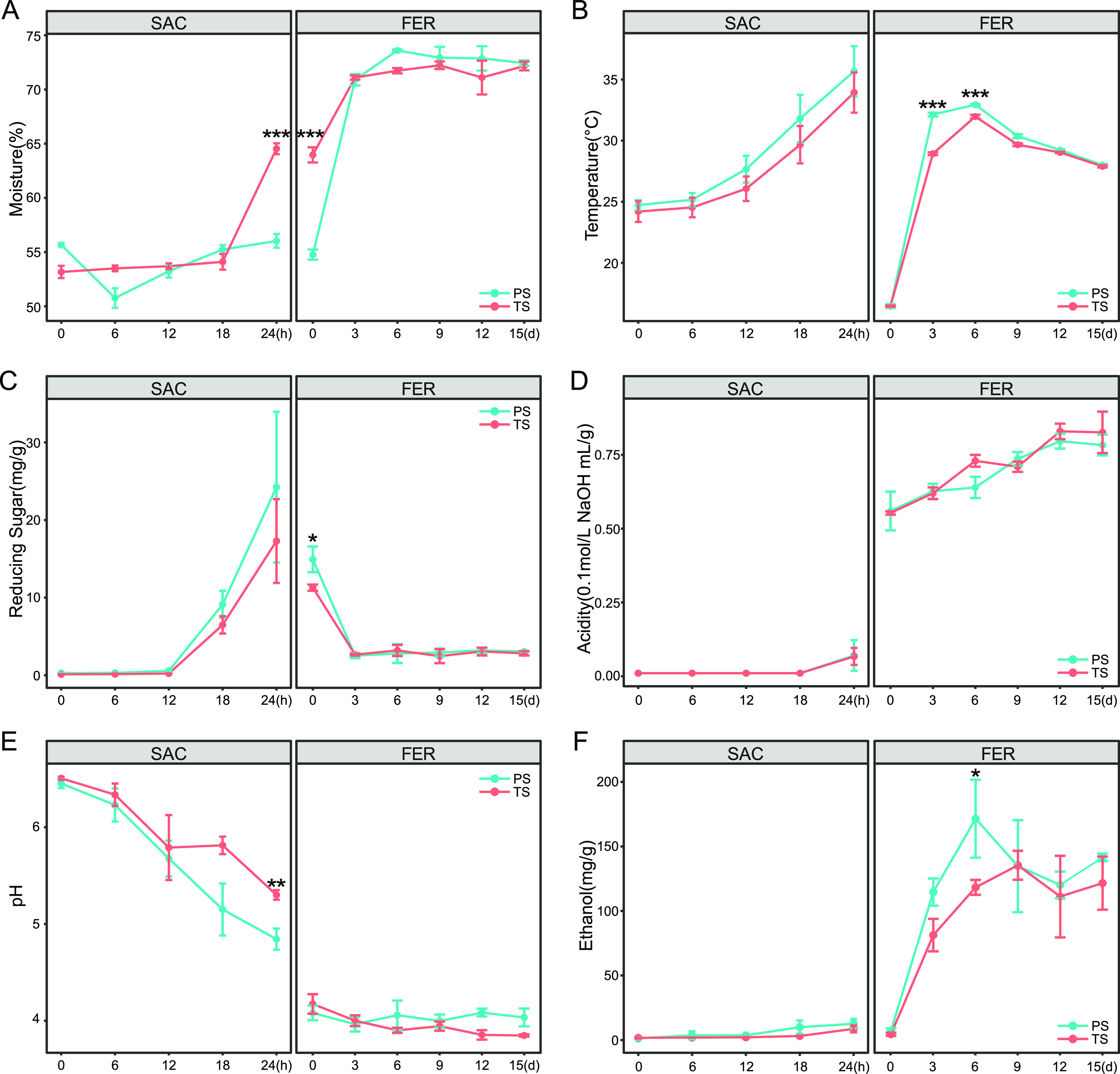
Dynamic changes of moisture (A), temperature (B), reducing sugar (C), acidity (D), pH (E), and ethanol (F) during the brewing process. The data are presented as means ± standard deviation (SD) (*n *= 3). A *t* test was conducted, with significant differences at three levels (*, *P* ≤ 0.05; **, *P* ≤ 0.01; ***, *P* ≤ 0.001).

The change trend of physicochemical parameters remained consistent between the two batches but differed between the two brewing stages (Fig. 2). Moisture, temperature, reducing sugar, and acidity were increased, while pH decreased during the middle and late stages of saccharification (Fig. 2A to D). The low concentration of ethanol confirmed that the saccharification stage was mainly in an aerobic environment, with anaerobic fermentation hardly occurring (Fig. 2F).

The physicochemical factors of fermented grains changed significantly (Fig. 2). In particular, there was a rapid increase in acidity with the addition of high-acidity distilled grains (Fig. 2D). Anaerobic fermentation also made the whole fermentation system gradually transition from aerobic to microaerobic then to anaerobic. According to the dynamics of fermented grain physicochemical factors, the fermentation stage was subdivided into the initial fermentation stage from day 0 to day 3 and the postfermentation stage from day 3 to day 15. In the initial fermentation stage, moisture, temperature, and ethanol were rapidly increased (Fig. 2A, B, and F), accompanied by a sharp consumption of reducing sugar (Fig. 2C). During the postfermentation stage, temperature and ethanol concentration were slowly decreased. Meanwhile, moisture and reducing sugar remained relatively stable, while acidity kept increasing during the postfermentation stage.

Since this experiment was conducted in a fully automatic production workshop with the external brewing conditions strictly under control, the dynamic changes in physicochemical factors were less likely to be caused by changes in the brewing environment. While research has shown that microbial metabolism could change the fermentation environment (23, 24), we speculated that the dynamic changes in physicochemical factors might be related to the succession and metabolism of microbial communities.

### Microbial composition during the brewing process.

A total of 6 starters, 30 saccharified grains, and 36 fermented grains were sampled from two separate groups for microbial composition analysis (Fig. 1). Amplicon sequencing of the 16S rRNA gene and ITS2 was performed to determine the bacterial and fungal composition, respectively, during the brewing process.

**FIG 3:**
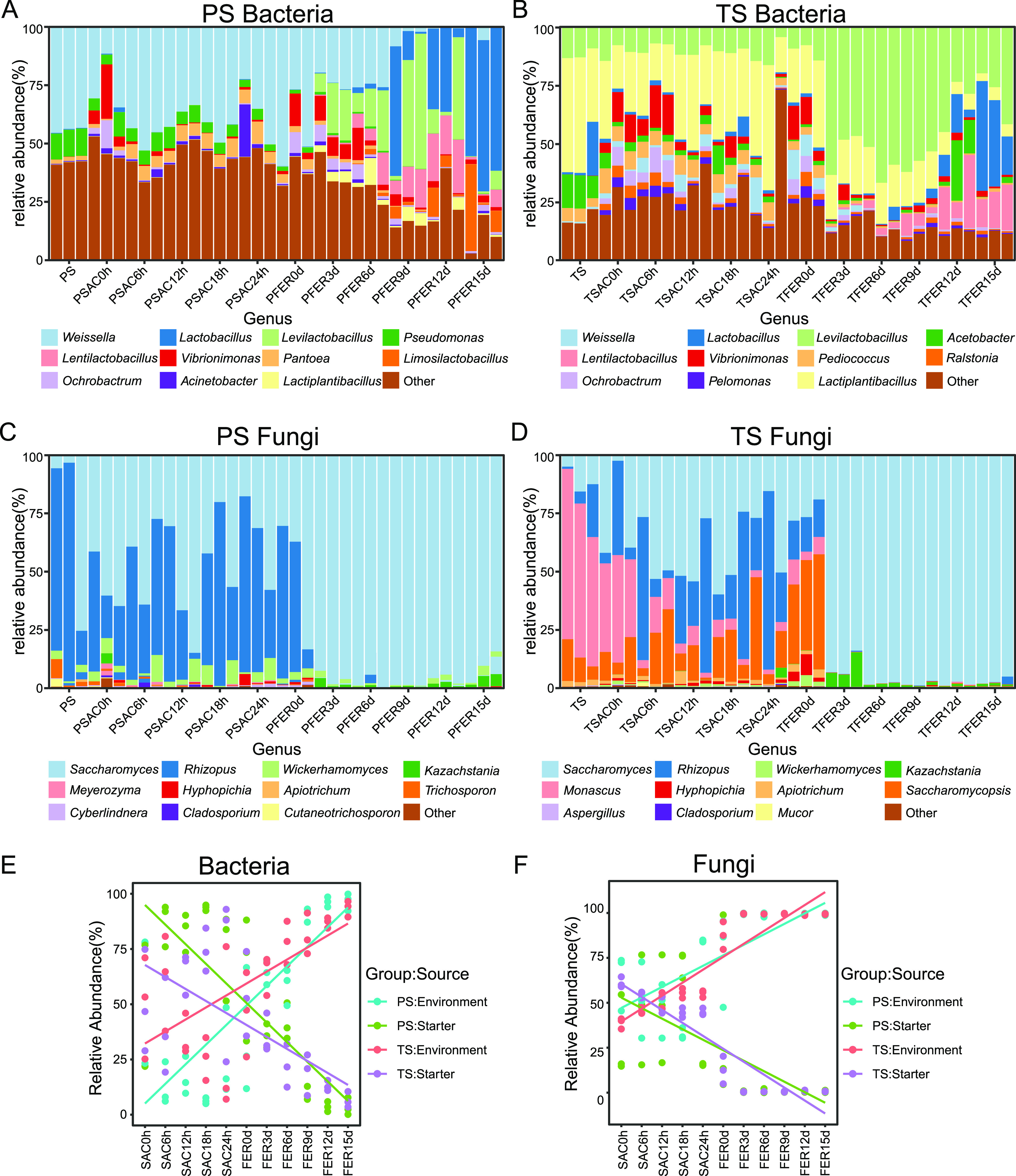
Compositions and sources of the microbial community during the brewing process. (A) Bacterial community composition of the PS batch. (B) Bacterial community composition of the TS batch. (C) Fungal community composition of the PS batch. (D) Fungal community composition of the TS batch. Only the top 11 genera with the highest abundance are shown in the bar plots. (E) Relative abundance of bacteria from different sources. (F) Relative abundance of fungi from different sources.

A total of 406 bacterial genera and 48 fungal genera were identified from all starters and grains (Fig. 3A to D). The microbial compositions of the two starters were significantly different. *Weissella* (44%), Pseudomonas (11.4%), and *Carnimonas* (8.4%) were dominant bacterial genera in PS (Fig. 3A), while lactic acid bacteria (LAB), including *Lactiplantibacillus* (43.2%), *Acetobacter* (12.8%), *Levilactobacillus* (11.9%), *Lactobacillus* (8.3%), *Pediococcus* (5%), and *Limosilactobacillus* (4.8%), were more abundant in TS (Fig. 3B). The dominant fungi in PS included Rhizopus oryzae (64.9%) and Saccharomyces cerevisiae (28%) (Fig. 3C; Fig. S2C), while those in TS consisted of Monascus purpureus (64.9%), Saccharomycopsis fibuligera (11.4%), Saccharomyces cerevisiae (11%), and *Rhizopus oryzae* (9.6%) (Fig. 3D; Fig. S2D).

The microbial community structures of saccharified grains were similar to those of starters. Some nonstarter-derived microorganisms with a relative abundance of less than 1% in the starters, such as *Vibrionimonas* (2.8% in the PS group and 6.3% in the TS group) and *Pantoea* (5.1% in the PS group and 3.1% in the TS group) were identified (Fig. 3A and B).

After stirring and acidification, the microbial compositions of fermented grains in the beginning of fermentation (FER0d) remained the same as those of saccharified grains. Unexpectedly, an identical succession pattern of the microbial compositions of fermented grains in both groups was discovered, regardless of the microbial compositions of saccharified grains. *Lactobacillaceae* (75.6% in the PS group and 86.8% in the TS group) and Saccharomyces cerevisiae (97.6% in the PS group and 96.3% in the TS group) were the dominant bacteria and fungi during fermentation, respectively, except in the beginning of the fermentation stage (Fig. 3A to D; Fig. S2).

This identical succession pattern of microbial compositions was irrelevant to the different saccharified microbial composition between the two batches, suggesting that the microorganisms involved in the fermentation stage might not be completely derived from the saccharified microbial community. Thus, we hypothesized that environmental microorganisms might be an important source of functional microorganisms. Further, we applied the SourceTracker algorithm to identify potential sources of functional microorganisms involved in the brewing process (25). We found that the saccharification microbiota was mainly derived from starter, while environmental microorganisms dominated the fermentation microbial community (Fig. 3E and F). Specifically, 79.5% and 59.3% of bacteria of the saccharification microbiota were derived from PS and TS, respectively, and 73.5% of bacteria in the PS group and 75.0% of bacteria in the TS group came from the fermentation environment. Similar results were found in the fungal community in which the PS and TS contributed about 50.0% and 48.0% of fungi to the saccharification community, respectively, while the environment contributed 95.2% and 98.5% of the fungal community in the PS group and the TS group, respectively, during the fermentation stage. These results confirmed our hypothesis that environmental microorganisms were an important part of the brewing microbial community and might be one of the potential factors to drive the succession of microbial communities, especially in the fermentation stage.

### Multistage succession pattern of microbial communities during the brewing process.

The stage changes in microbial compositions indicated that multistage succession of microbial communities occurred during the brewing process. The succession of microbial communities is usually manifested by two distinct patterns: turnover in which some species were replaced by others over time, namely, species replacement, and nestedness where one community acts as a subset of another community, including species gain or species loss (26).

**FIG 4:**
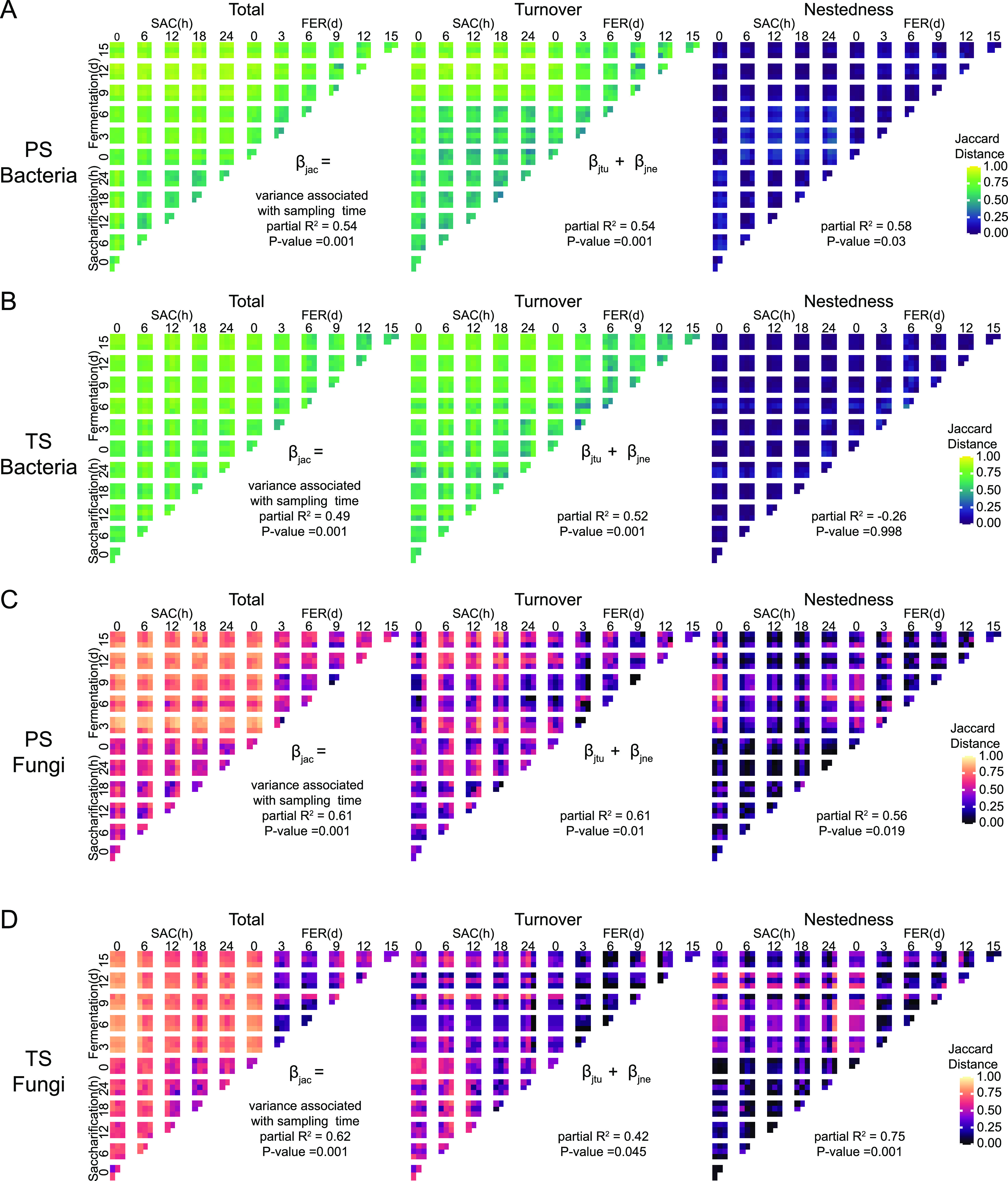
Multistage community succession patterns during the brewing process. Jaccard distance matrix of the bacterial community in the PS batch (A) and TS batch (B) and that of the fungal community in the PS batch (C) and TS batch (D). Jaccard distance is partitioned into the component of turnover (species replacement) and nestedness (species loss). Lighter colors represent pairwise samples with less shared ASVs. β_jac_ is the total Jaccard dissimilarity. β_jtu_ is the turnover component of Jaccard dissimilarity. β_jne_ is the nestedness component of Jaccard dissimilarity.

To assess the relative contribution of species replacement and species gain/loss to the functional microbial community successions during the brewing process, Jaccard distance (total beta diversity) was partitioned into two parts, turnover and nestedness (Fig. 4). A similar pattern of bacterial community succession was observed during the whole brewing process in the two groups (Fig. 4A and B), in which turnover was the dominant community succession pattern, and more than 50% of the observed changes from turnover were related to sampling time. Therefore, replacement of starter bacteria with environmental bacteria was the main succession pattern of the bacterial community during the baijiu brewing process.

Surprisingly, the succession pattern of fungal communities was quite different from that of bacterial communities (Fig. 4C and D). Although turnover explained almost all the fungal community changes in the saccharification stage and partial changes in the initial fermentation stage, the remaining considerable proportion of changes in the initial fermentation stage were explained by nestedness. The decrease in the diversity of the fungal community from the saccharification stage to the fermentation stage suggested that species loss was the dominant nestedness pattern in this stage transition (Fig. S3B). Approximately 56% of nestedness in the PS group and 75% of nestedness in the TS group were related to the temporal succession of the fungal community, indicating the importance of nestedness for community succession. Based on the above results, we concluded that turnover and nestedness (species loss) codominated the succession of fungal communities during the brewing process.

Overall, although they were in the same ecosystem, bacterial and fungal communities exhibited different succession patterns during the brewing process, suggesting that the ecological processes affecting the community succession patterns as well as their determinants might not be the same.

### Determinants of microbial community succession during the brewing process.

It has been reported that multiple factors, including temperature, oxygen, moisture, pH (acidity), and ethanol, affect the microbiota during liquor brewing (21). To investigate the determinants of microbial community succession during *Xiaoqu* liquor brewing, a correlation analysis between physicochemical factors and microbial diversity was conducted (Fig. S4). We found that physicochemical factors strongly influenced the bacterial community but not the fungal community (Fig. S4A and B). Specifically, the bacterial alpha diversity indices were negatively correlated with temperature, reducing sugar, and ethanol but were positively correlated with pH (Fig. S4A). However, the alpha diversity indices of the fungal community exhibited no significant correlation with these physicochemical factors (Fig. S4B). The similar results were observed in distance-based redundancy analysis (dbRDA) ordination. The top two dbRDA axes explained 22.87% of the bacterial community structure variation (Fig. S4C) but only 7.60% of the fungal community structure variation (Fig. S4D).

**FIG 5:**
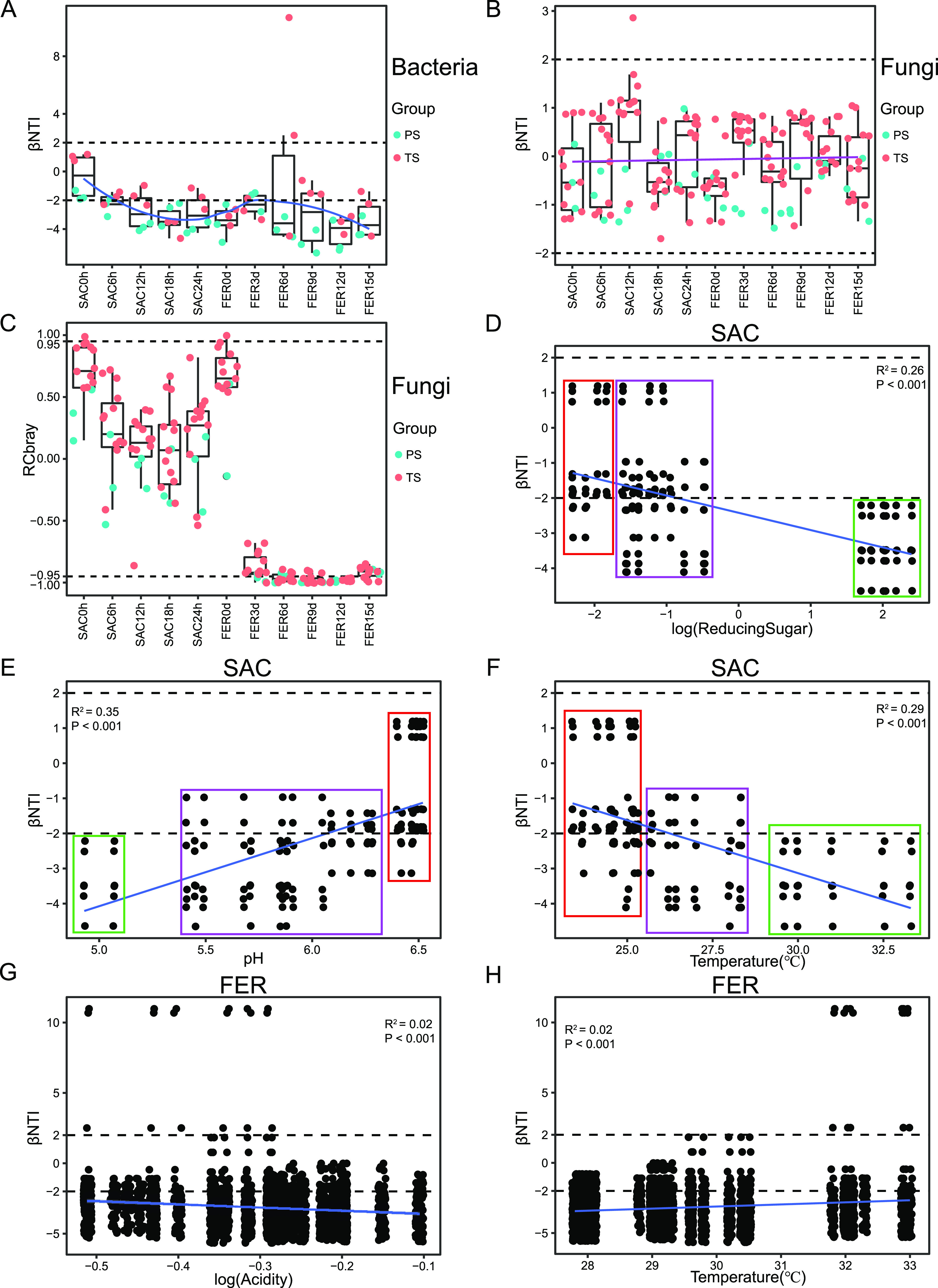
Ecological processes that generate the community succession patterns and corresponding determinants during the brewing process. (A) βNTI results of the bacterial community. (B) βNTI results of the fungal community. (C) RC_bray_ results of the fungal community; βNTI > 2, variable selection; βNTI < −2, homogeneous selection; |βNTI| < 2, stochastic process; RC_bray_ > 0.95, dispersal limitation; RC_bray_ < −0.95, homogenizing dispersal; |RC_bray_| < 0.95, drift. βNTI is calculated within, but not between, each sampling time point. RC_bray_ is calculated only when pairwise βNTI is insignificant (|βNTI| < 2). A linear regression model indicates that log-transformed reducing sugar (D), pH (E), and temperature (F) are correlated with a deterministic process during the saccharification stage, while log-transformed acidity (G) and temperature (H) are correlated with a deterministic process in the postfermentation stage.

To further explore the determinants of the succession patterns of microbial communities, we implemented a statistical framework based on a null model to infer the ecological processes affecting the microbial community assembly (Fig. 5A to C) (27). As shown in Fig. 5A, the bacterial community assembly model exhibited an obvious primary succession-disturbance-secondary succession pattern. The initial stage of bacterial community assembly was predominated by a stochastic process (−2 < beta mean nearest taxon index [βNTI] < 2), indicating that the bacterial community composition was randomly formed and unaffected by the external environment. With the establishment of the initial microbial community, the saccharification of raw materials by microorganisms affected and further changed the brewing environment. Therefore, environmental selection (homogeneous selection; βNTI < −2) became increasingly important during the saccharification stage and led to a close phylogenetic relationship between the bacteria within the community. Homogeneous selection usually occurred when changes in one or more selective factors led to accumulation of the environmental selection pressure, resulting in the increased number of microorganisms with strong adaptability to the new environment (28). Further regression analysis showed that pH, temperature, and reducing sugar were the driving factors for the succession of bacterial communities in the saccharification stage (Fig. 5D to F). The increasing reducing sugar and the rising temperature along with the decreasing pH led to a transition of the ecological process from weak selection and a stochastic process (low reducing sugar, low temperature, and high pH) to a mixture of a stochastic process and homogeneous selection (moderate reducing sugar, temperature, and pH) and finally to homogeneous selection (high reducing sugar, high temperature, and low pH). In the initial fermentation stage, homogeneous selection still governed the bacterial community assembly (βNTI < −2), but it trended toward a stochastic process (βNTI close to 0), which might be due to the mechanical homogenization of the grains during the transition from saccharification to fermentation. On the one hand, mechanical stirring of the grains, as an external disturbance, removed the strong selective pressure developed from the saccharification. On the other hand, grain acidification and the lack of oxygen induced a new selection pressure, resulting in a new homogeneous selection to the bacterial community in fermentation. Acidity and temperature were the determinants for the assembly of bacterial communities in the fermentation stage (Fig. 5G and H). Taken together, acidity, reducing sugar, and temperature were the determinants of bacterial community assembly during the brewing process.

Fungal community assembly during liquor production, on the contrary, was governed by a stochastic process rather than strong ecological selection (Fig. 5B). Further decomposition of the stochastic process revealed that ecological drift (−0.95 < Raup-Crick Bray-Curtis index [RC_bray_] < 0.95) dominated the fungal community assembly during the saccharification stage, while homogenizing dispersal (RC_bray_ < −0.95) governed it in the fermentation stage (Fig. 5C). In the initial fermentation stage, RC_bray_ values close to −0.95 could be considered a mixing effect of homogenizing dispersal, and ecological drift affected the fungal community succession. Meanwhile, homogenizing dispersal dominated the fungal community assembly during the postfermentation stage (RC_bray_ < −0.95). Therefore, homogenizing dispersal governed the fungal community assembly throughout the fermentation stage. Accumulation of acids and ethanol and reduction of oxygen could affect the fungal community, especially filamentous fungi (21). Since acidity, pH, and ethanol were not significantly correlated with fungal diversity (Fig. S4B), we speculated that oxygen might be an important factor affecting fungal community succession in the fermentation stage. We inferred that the decrease of oxygen caused the disappearance of fungi such as *Rhizopus oryzae*, *Monascus purpureus*, and Saccharomycopsis fibuligera, while Saccharomyces cerevisiae adapted to the high-acidity and hypoxic environment during the fermentation stage. Great adaptability to this environment led to the high-level dispersal of Saccharomyces cerevisiae (29). Thus, the entire fungal community was homogenized, and Saccharomyces cerevisiae dominated the entire fermentation fungal community.

### Profiles of volatile metabolites during the brewing process.

**FIG 6:**
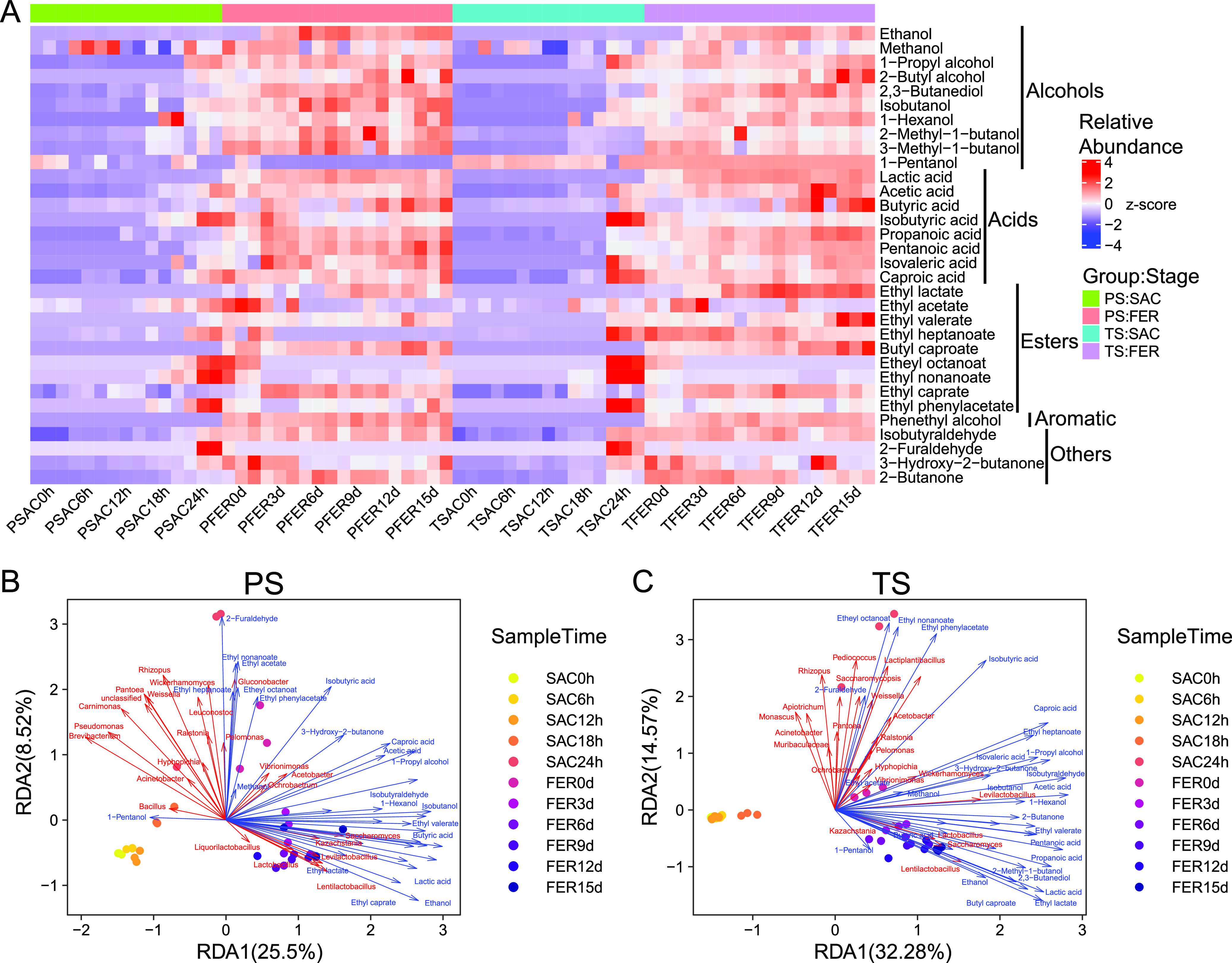
(A) Heat map of relative abundance of metabolites in each stage. Redundancy analysis (RDA) of dominant microbes (red) at genus levels and flavor compounds (blue) in the PS group (B) and the TS group (C). Only relative abundances of microbes above 0.1% were considered to the dominant microbes. Flavor compounds were Z-score transformed.

Changes of microbial community composition may influence the expression of metabolic functions (30, 31). A total of 66 grains and 6 fresh liquors from two groups were collected for metabolic components analysis. Thirty-two metabolic components were identified, including 10 alcohols, 8 acids, 9 esters, 1 aromatic, and 4 others (Fig. 6A). Principal-component analysis (PCA) showed that metabolites exhibited dynamical changes over the brewing process, with the PC1 axis contributing 52.5% of the total variation. Metabolites were clustered into two groups corresponding to two different brewing stages (Fig. S5) (permutational multivariate analysis of variance [PERMANOVA] *R*^2^ = 0.44096, *P = *0.001). Metabolites from two groups with different starters exhibited no differences during the saccharification stage (PERMANOVA *R*^2^ = 0.04273, *P = *0.264) but displayed a slight difference during the fermentation stage (PERMANOVA *R*^2^ = 0.09528, *P = *0.001).

Most of the metabolites were produced during the fermentation stage (Fig. 6A), while some esters were produced at the end of saccharification, which might be the result of excessive oxygen consumption in the late stage of saccharification. More alcohols were produced in the PS group, while more acids and esters were produced in the TS group (Fig. S6). Similar results were also observed in the analysis of the fresh liquor (Table S3). The fresh liquor produced from the PS group contained more alcohols, including methanol, isobutanol, 3-methyl-1-butanol, and fusel oils, while the fresh liquor produced from the TS group contained more ethyl acetate. Considering that the microbial compositions of the two groups were similar in the fermentation stage, we speculated that the different microbial community structures in the saccharification stage might contribute to such differences in metabolites directly and indirectly. Thus, a redundancy analysis (RDA) was further used to analyze the correlations between microorganisms and metabolites (Fig. 6B and C). Overall, the top two RDA axes explained 34.02% and 46.85% of the variation of metabolites in the PS group and TS group, respectively, suggesting a strong correlation between microbial communities and flavor compounds. As expected, despite the fact that most of the flavor compounds, including alcohols (ethanol, isobutanol, 1-propyl alcohol, and 1-hexanol), acids (lactic acid, acetic acid, and butyric acid), and some esters (ethyl lactate, ethyl valerate, and ethyl caprate), were positively correlated with the dominant genera in the fermentation stage, such as *Saccharomyces*, *Kazachstania*, *Lactobacillus*, *Lentilactobacillus*, and *Levilactobacillus*, in both groups, the metabolism of several esters, such as etheyl octanoate, ethyl nonaoate, ethyl phenylacetate, and ethyl acetate, were positively correlated with those genera predominant in the saccharification stage, including *Rhizopus*, *Gluconobacter*, *Bacillus*, Pseudomonas, and *Weissella* in the PS group and *Saccharomycopsis*, *Lactiplantibacillus*, *Pediococcus*, *Monascus*, and *Acetobacter* in the TS group.

## DISCUSSION

With the development of next-generation sequencing technology, modern “omics” approaches have been broadly applied to evaluate the ecological and functional dynamics of fermented foods (32). In this study, we combined amplicon sequencing with gas chromatography-mass spectrometry (GC-MS) to explore the temporal succession trajectories and community assembly patterns of the microbial community in the two-stage brewing process of *Xiaoqu* liquor and its influence on the metabolism of flavor compounds.

A βNTI value close to 0 at the beginning of saccharification indicated that microorganisms from the starters randomly formed the initial microbial community (Fig. 5A and B). It seemed that the initial saccharification environment showed no strong selection forces. However, after initial microbial community establishment, homogenous selection became increasingly important for bacterial community assembly with increasing environmental selection pressure. The rapid changes of fermentation parameters induced by microbial metabolism, known as niche modification, further mediated the environmental filtration of bacterial communities. That is to say, the early-colonized microbiota exerted priority effects. Reducing sugar, pH, and temperature were the determinants driving the bacterial community assembly in the saccharification stage (Fig. 5D to F). This result is consistent with previous study findings (21). As individual microorganisms have different adaptability to environmental pH, it has been reported that pH has a great influence on the composition of microorganisms in many natural ecosystems (33–35). It has also been found that temperature affects the functional microbial community structure in the brewing process of liquor in both the short (23) and long term (17). Meanwhile, most bacteria rely on reducing sugar produced by fungal degradation of starch as a carbon source for proliferation and metabolism in the liquor brewing ecosystem (36). Previous studies have found that the composition of reducing sugars produced by different raw materials during liquor brewing is different, further leading to the structural differences in bacterial communities (37). The succession of the fungal community in the saccharification stage was governed by a stochastic process. Unlike bacteria, the dominant fungi during saccharification, especially *Rhizopus*, *Monascus*, and *Saccharomycopsis*, could secrete amylase and protease to degrade sorghum starch and produce flavor compounds, such as organic acids (36, 38). In our study, the proper amount of oxygen in the open saccharification environment allowed the fungal community to obtain sufficient energy from sorghum starch for growth. Therefore, the fungal community might not be affected by environmental selection pressure. We speculated that the fungal community, as primary producers, might change the fermentation environment by metabolizing organic acids and create new niches by producing reducing sugars. Under environmental selection pressure, new niches selectively recruit environmental bacteria (39), thus indirectly promoting the succession of bacterial communities, indicating that there might show succession regulation between different microbial domains in a multidomain ecosystem. Our speculation is in line with one previous report on a microbial succession where viral predation makes bacterial community assembly transition from homogeneous selection to random assembly under hypersaline stress in the shale ecosystem (40). Additionally, a symbiotic relationship between *Lactobacillus* and *Saccharomyces* has been observed in the liquor fermentation process (41). All these findings suggest a possible coordinated relationship between different microbial domains.

Macro events in natural ecosystems, such as wildfire (5), permafrost thaw (42, 43), flood (44), drought (45), and soil salinity (46), have been reported to contribute considerably to the change and secondary succession of microbial communities. As expected, disturbances, including mechanical stirring, grain acidification, and oxygen reduction, in the fermentation stage caused substantial changes in microbial diversity (Fig. S3 in the supplemental material), composition (Fig. 3), biomass (Fig. S7), and fermentation environment properties (Fig. 2). We also observed a rapid increase in metabolic components after the disturbances (Fig. 6A), suggesting that the disturbances might also change the metabolism of the microbial community. Collectively, these results indicated that disturbances changed the whole fermentation microbial ecosystem directly and indirectly. Our data indicated that disturbances reshaped the secondary succession by destroying the microbial ecosystem generated from the primary succession. The microbial compositions of both batches were almost identical in the postfermentation stage despite the different microbial composition in the saccharification stage (Fig. 3A to D). The fermentation microbial composition only depended on the environmental microorganisms from the regional species pools (Fig. 3E and F), indicating that the environmental selection pressure generated from saccharification was completely removed. These results jointly suggested that microbial communities might have diverse responses to different degrees of external disturbances. One previous study has confirmed that the soil microbiome under different degrees of vegetation coverage has significantly different responses to wildfire disturbances (47), which supports our findings.

Microorganisms from different domains might have different succession patterns (47) and different response patterns to external environmental changes (48). We found significant differences in the microbial succession patterns and their responses to disturbances between bacterial and fungal communities during the brewing process (Fig. 4). The dominant succession pattern of the bacterial community was turnover while that of the fungal community was a combination of turnover and nestedness. In the saccharification stage, a deterministic process (Fig. 5A) and stochastic process (Fig. 5B) governed the bacterial and fungal community assembly, respectively. Although the assembly of both communities after disturbance was dominated by environmental selection in the fermentation stage, the determinants of their environment selections were different, where acidity and oxygen were the determinants for bacterial and fungal communities, respectively. Additionally, dynamic changes of the microbial biomass after disturbance were also different between the two communities (Fig. S4), further confirming that bacterial and fungal communities had different succession patterns. Such a difference in succession pattern might be attributed to the different traits, including preference for different carbon sources, adaptability to acid stress, and demands for oxygen between bacteria, filamentous fungi, and yeast in the brewing environment (36, 49). Sorghum starch cannot be utilized by most of the bacteria and yeasts; thus, it needs to be further hydrolyzed into reducing sugars through amylase and glucoamylase, which are normally secreted by filamentous fungi (50, 51). The cooccurrence of two distinct succession patterns in the fungal community during the brewing process might be attributed to the different phenotypes between yeast and filamentous fungi. Some microorganisms that were inefficient in certain specific abilities might be more adaptable to disturbances and environmental stresses than those specialist microorganisms (52–54). In spite of a short life span, these organisms are able to spread and reproduce quickly without a high demand on living environment; therefore, they are more likely to benefit from drastic changes in the external environments (55). Fungi such as *Rhizopus oryzae*, *Monascus purpureus*, and Saccharomycopsis fibuligera in this study are specialized in secreting hydrolytic enzymes to use starch (36, 56). The sufficient oxygen and starch in the saccharification stage were particularly suitable for the survival of these specialist fungi. However, oxygen reduction and high acidity stress in the initial fermentation stage might cause the extinction of these fungi, thus leading to species loss (36). Meanwhile, the generalist Saccharomyces cerevisiae derived from starters or the environment might be more adapted to the environment in the fermentation stage since it could tolerate higher acid and ethanol than filamentous fungi (57, 58). It could also form a mixed-species biofilm in liquor brewing to increase the tolerance to environmental stress (41). Thus, the fungal community was reshaped, and Saccharomyces cerevisiae dominated the new fungal community. These trait-related environmental factors tend to affect the structure of microbial communities (59); however, the current results were not sufficient to explain the interdomain relationships. Therefore, further research needs to be performed. Our results showed that fermented food ecosystems could be promising experimental models for multidomain microbial community ecology research.

The flavor compounds in fermented foods were key components determining consumer preference and control targets in food fermentation production (60). These flavor compounds are often metabolized by complex microbial communities; therefore, flavor compounds vary with changes in microbial communities (17). Despite the fact that two groups shared similar compositions of functional microbial communities in the fermentation stage, metabolites of grains and fresh liquor still exhibited differences between the two groups (Fig. S5, Table S3). Functional microorganisms involved in the liquor fermentation stage could exhibit diverse metabolism ability under different environments (61). For instance, oxygen levels (62), types of the carbon source (63), the external glucose concentration (64), or even symbionts (61) have been reported to regulate the metabolism of Saccharomyces cerevisiae, which is the main functional fungus in the fermentation stage. It has been reported that *Rhizopus oryzae*, which was the dominant fungus in the saccharification stage of the PS group, has a strong starch degradation ability (22, 65). Higher reducing sugar produced by *Rhizopus oryzae* in the PS group stimulated both growth and metabolism of Saccharomyces cerevisiae, resulting in higher yeast abundance and more alcohols in the fermentation stage (Fig. 2; Fig. S7). Meanwhile, *Monascus purpureus* and Saccharomycopsis fibuligera, two major fungi in the saccharification stage of the TS group, have been found to have strong abilities to secret several esterases (66–68). Thus, the higher ester concentrations in the TS group could be attributed to those saccharifying fungi, since the esterases could be continuously effective even if the fungi were extinct in the fermentation stage. These results indicated that the functional microbes in the saccharification stage were not only the saccharification agents, but also influenced the metabolisms of flavor compounds directly and indirectly. This also suggested that regulating the saccharification microbial community is an option to improve the quality of *Xiaoqu* liquor. By examining the succession patterns and determinants of the functional microbial communities involved in food fermentation, it is possible to artificially control microbial community succession to optimize the production of flavor compounds (19). Our investigation of unique two-stage microbial community succession revealed that *Xiaoqu* liquor production could be regulated by controlling disturbance. By delaying the disturbance, we could enhance the sorghum saccharification by *Rhizopus oryzae* to increase liquor yield or promote the esterase secretion of *Monascus purpureus* and Saccharomycopsis fibuligera to increase the synthesis of esters, the main flavor substances in Chinese liquor.

Overall, our research characterized the microbial composition, fermentation system properties, and flavor metabolism in the two-stage brewing of Xiaoqu Chinese liquor. Furthermore, we analyzed the succession patterns and ecological assembly processes of the microbial community in the brewing process. This unique two-stage brewing process also provided us an opportunity to examine the microbial communities’ responses to external disturbances, which was valuable for microbial community ecology research. Combined with flavor compound analysis, this study also provided a new perspective on the influence of microbial community succession on the metabolic functions of microbial communities, which was beneficial to the modern industrial production of fermented foods. The results obtained in this study were also valuable for the research of microbial community ecology in other ecosystems.

## MATERIALS AND METHODS

### Experimental design and sample collection.

Two types of brewing starter *Xiaoqu*, pure starter (PS) and traditional starter (TS), were manufactured at Jing Brand Maopu No. 3 distillery (Daye City, Hubei Province, China) and Jing Brand Maopu No. 1 distillery (Huangshi City, Hubei Province, China), respectively. For PS, three fungal strains, one *Rhizopus oryzae* G1 (China General Microbiological Culture Collection Center [CGMCC] no. 7.13) and two Saccharomyces cerevisiae Y1 (CGMCC no. 7.14) and Y2 (CGMCC no. 7.15) were inoculated into the wheat bran. After a 3-day maturation, the obtained cultures were crushed into powders. For TS, soil, rice, rice bran, and a previous batch of TS powder were crushed, mixed with sterile water, shaped into small balls, and placed in a special culture room. After 2 months of maturation, the obtained starter balls were also ground into powder. Both starter powders were then delivered to the Jing Brand Maopu No. 3 distillery for subsequent brewing experiments.

Three batches of brewing experiments were continuously performed in November 2019. In each batch, two experimental brewing processes inoculated with two different starters, PS and TS, were set up simultaneously (Fig. 1). First, sorghum was soaked in hot water, steamed, and cooled. Steamed sorghum was then mixed with 1% starter powder. The mixtures were then delivered to the saccharification chambers, where the temperature was not less than 25°C, and stacked into shallow layers for 24 h of aerobic saccharification. After saccharification, the saccharified grains were fully stirred, mixed with half of the previous batch of high-acidity distilled grains, and recooled. The mixed grains were then transferred into the stainless-steel fermenters and covered with plastic films to isolate oxygen. Fermenters were placed in a fermentation chamber with a temperature not higher than 20°C for anaerobic fermentation. After 15 days of fermentation, two groups of fermented grains were distilled separately for fresh liquor.

Experimental samples, including starter powder, saccharified grains, fermented grains, and fresh liquors, were collected from the three batches in two groups. In each batch, saccharified grains were collected at 0, 6, 12, 18, and 24 h of the saccharification stage. At each time, three saccharified grains were randomly collected from the saccharification box and further mixed into one sample in each group. Fermented grains were sampled at 0, 3, 6, 9, 12, and 15 days of the fermentation stage. At each sampling time, fermented grains were collected from the center and four corners of the fermenter’s middle layers in each group, which were further mixed into one sample. Due to the premixing in the production process, only one starter powder and fresh liquor were collected in each group in each batch. Hence, a total of 15 saccharified grain samples, 18 fermented grain samples, 3 starter powder samples, and 3 fresh liquor samples were collected from three batches in each group. Grain samples were named by the combined information of the starter group (P for PS group, T for TS group), brewing stage (SAC for saccharification stage, FER for fermentation stage), and sampling time points and batch numbers. For example, TFER3d1 represented the fermented grains sampled at day 3 from batch 1 in the TS group. Starters and grains were immediately frozen with liquid nitrogen and stored at −20°C for further experiments.

### Analyses of physicochemical parameters and volatile compounds.

Moisture of all 66 grains was determined by the weight loss of 10 g of sample dried at 135°C for at least 2 h (37). Grain pH was assessed in ultrapure water at a ratio of 1:5 (wt/vol) using a Mettler Toledo FiveEasy Plus pH meter (69). Temperature was measured in real time by inserting six thermometers into each group of saccharification layers and stainless-steel fermenters (37). Reducing sugar was quantified by the feline method (70). Acidity was analyzed by an acid-based titration method (71). Consumption of 0.1 M NaOH by 1 g of sample was defined as one unit of acidity. To evaluate the concentration of ethanol and organic acids in grains, 5 g of sample was mixed with 20 ml of ultrapure water, ultrasonicated at 0°C for 30 min, and centrifuged at 8,000 × *g* for 5 min at 4°C to obtain the supernatants. High-performance liquid chromatography (HPLC) was used to determine the concentration of ethanol and organic acids in the supernatants. Volatile compounds in fresh liquors and supernatants were quantified using headspace solid-phase microextraction gas chromatography-mass spectrometry (HS-SPME-GC-MS) according to the method reported by Gao et al. (72).

### DNA extraction and amplicon sequencing.

Grains and starter powders were used for amplicon sequencing. Total DNA was isolated using a DNeasy PowerSoil Pro kit (Qiagen Inc, Hilden, Germany) according to the manufacturer’s instructions. A FastPrep-24 bead beating system was used to homogenize samples before DNA extraction. The quantity and quality of DNA were determined using a NanoDrop ND-2000 spectrophotometer (NanoDrop Technologies, Wilmington, DE, USA). DNA was stored at −80°C before further analysis.

For bacteria, the V4 hypervariable region of the 16S rRNA gene was amplified with primers 515F (Parada) and 806R (Apprill) (73, 74). For fungi, internal transcribed spacer 2 (ITS2) was amplified with primers fITS7 and ITS4-Fun (75, 76). PCR products were verified by agarose gel electrophoresis and purified with Agencourt AMPure XP beads (Beckman Coulter, Brea, CA, USA). DNA concentrations of purified amplicon libraries were quantified with a PicoGreen dsDNA assay kit (Invitrogen, Carlsbad, CA, USA). After the quantification step, amplicon libraries were pooled in equal amounts and then loaded on an Illumina Novaseq 6000 Platform for PE250 sequencing (Illumina, San Diego, CA, USA).

### Quantitative real-time PCR.

To estimate the abundance of bacteria and fungi during the brewing process, quantitative real-time PCR (qPCR) was performed with the same primer pairs used in amplicon sequencing on a QuantStudio5 system (Applied Biosystems, Foster City, CA, USA). All reactions were performed in 20-μl volumes containing 1× ChamQ SYBR qPCR master mix (Vazyme, Nanjing, China), 0.4 μM primers, and about 20 ng of total genomic DNA. The following thermal conditions were used: predenaturation at 95°C for 3 min, followed by 40 cycles of denaturation at 95°C for 10 s, annealing at 54°C for 30 s, and elongation at 72°C for 30 s, as recommended by the manual.

### Bioinformatics analysis.

Raw sequences were mainly processed based on QIIME2 (2020.6) (77). Briefly, the raw sequences were demultiplexed using q2-demux, followed by primer removing with q2-cutadapt (78). Quality filtering, denoising, and chimera removal were performed by q2-dada2 (79). The amplicon sequence variants (ASVs) generated from the DADA2 pipeline were blasted against the nonredundant (nr) database using blastx (E value ≤ 10^−5^) (blast+ version 2.9.0), and nontarget sequences were removed based on their annotation titles. Singletons and ASVs that were only found in one sample were further removed. Taxonomy was assigned to ASVs using the naive Bayes taxonomy classifier against the pretrained Silva 138.1 NR99 reference database and the developer version of Unite 8.2 fungal dynamic database, respectively (80–82). Additional taxonomy assignment was performed using the function ‘addSpecies’ in the R package dada2 (1.16) (83). All bacterial ASVs were aligned with mafft, and a phylogenetic tree was constructed using q2-phylogeny (84, 85). Due to the poor quality of ITS multiple sequence alignments, a hybrid tree based on the Silva 138 18S database and Unite 8.2 fungal dynamic database was constructed using q2-ghost-tree (86).

### Statistical analysis.

Statistical analyses were mainly conducted by QIIME 2 (2020.6) and R (3.6.3). Data processing and visualization were performed through the R metapackage tidyverse (1.3.0) (87). Kruskal-Wallis rank sum tests, *t* tests, and Wilcoxon rank sum tests were performed through the functions “t.test”, “kruskal.test”, and “wilcox.test” in the package “stats” (3.6.3). Before analysis, samples were rarefied to uniform depth based on the lowest sample sequence to eliminate the influence of different sequencing depth. Alpha diversity indices (Shannon, Pielou’s evenness, observed species, and Faith’s phylogenetic diversity [PD]) were calculated using q2-diversity (88). Alpha rarefaction curves were performed based on observed species index to evaluate whether the sequencing depth was sufficient. Beta diversity metrics (Jaccard dissimilarity, Bray-Curtis dissimilarity) as well as nonmetric multidimensional scaling (NMDS) were evaluated using the R package vegan (2.5-6) (89). To examine the effect of physicochemical parameters on the microbial community variation, distance-based redundancy analysis (dbRDA) was executed based on Bray-Curtis dissimilarity using the function “capscale” in vegan after physicochemical parameters were standardized using the Z-score transformation method with the function “decostand” in vegan (90). Volatile compounds (Z-score transformed) were visualized through ComplexHeatmap (2.2.0), and a principal-component analysis (PCA) was conducted using the function “rda” in vegan. Redundancy analysis (RDA) was used to evaluate the correlations between the microbial community and volatile compounds.

SourceTracker was performed to identify the potential sources of the microorganisms (25). To evaluate the relative contribution of turnover (species replacement) and nestedness (species gain/loss) to the microbial community succession, Jaccard dissimilarity was partitioned using the R package betapart (1.5.2) (91) followed by a PERMANOVA test to measure the changes related to sampling time (92).

### Null model analysis.

Several different ecological processes might govern community assembly of the functional microbial communities during the brewing process in the context of the determinism-versus-stochasticity dichotomy: homogeneous selection, heterogeneous selection (variable selection), homogenizing dispersal, dispersal limitation, and drift. Homogeneous selection and heterogeneous selection belong to the deterministic process, a niche-based process that shapes community structure due to differences between diverse microorganisms. The effects of abiotic environmental filtering and biotic interactions were generally classified into the deterministic process. Homogeneous selection is defined as homogeneous environmental conditions that lead to similar structures among communities, while heterogeneous selection means heterogeneous environments lead to different community structures. However, homogenizing dispersal and dispersal limitation were classified into dispersal, which is a process where individual microorganisms move from one location and successfully colonize another location. Homogenizing dispersal means that high-rate dispersal of microorganisms caused similar structures among communities, while dispersal limitation indicates that weak mobility of microorganisms caused more dissimilar structures between communities. Drift is the process where community heterogeneity was only caused by the inherent stochastic processes of birth, death, and reproduction (29).

To further evaluate the balance between the deterministic process and stochastic process on governing the functional microbial communities during the brewing process, a null model analysis was performed to pairwise compare the beta mean nearest taxon index (βNTI) and Raup-Crick Bray-Curtis index (RC_bray_) of the microbial community within one sampling time rather than across different sampling times during the brewing processes, as described by Stegen et al. (27, 28). Briefly, βNTI was used to evaluate whether the phylogenetic similarity between the two samples was significantly lower or higher than theoretical expectation. If the phylogenetic similarity was lower than theoretical expectation (βNTI > 2), it could be speculated that variable selection dominated the community succession. Phylogenetic similarity higher than expectation (βNTI < −2) indicated the dominance of homogeneous selection. The approximation to the theoretical expectation (−2 < βNTI < 2) meant that the community assembly was driven by a stochastic process rather than a deterministic process. In the case of −2 < βNTI < 2, RC_bray_ was further used to determine the type of stochastic process. If RC_bray_ was greater than 0.95, the difference between communities was due to dispersal limitation. An RC_bray_ value of less than −0.95 meant that the difference between communities was lower than the theoretical expectation and that the community succession was affected by the homogenizing dispersal. If −0.95 < RC_bray_ < 0.95, community heterogeneity was caused by ecological drift (random proliferation or death of microorganisms, weak dispersal, weak selection). Generalized linear models were further fitted to the physicochemical parameters and βNTI to infer the determinants of the community succession dominated by the deterministic process.

### Data availability.

All sequencing reads generated in this study are publicly available through the SRA database under accession number PRJNA661729.
